# Phthalates exposure and pubertal development in a 15-year follow-up birth cohort study in Taiwan

**DOI:** 10.3389/fendo.2023.1065918

**Published:** 2023-05-23

**Authors:** Pen-Hua Su, Jing-Yang Huang, Shu-Li Julie Wang, Hua-Pin Chang

**Affiliations:** ^1^ Department of Pediatrics, Chung Shan Medical University Hospital, Taichung, Taiwan; ^2^ School of Medicine, Chung Shan Medical University, Taichung, Taiwan; ^3^ Department of Medical Research, Chung Shan Medical University Hospital, Taichung, Taiwan; ^4^ Institute of Medicine, Chung Shan Medical University, Taichung, Taiwan; ^5^ National Institute of Environmental Health Sciences, National Health Research Institutes, Miaoli, Taiwan; ^6^ Department of Public Health, National Defense Medical Center, Taipei, Taiwan; ^7^ Department of Nursing, Asia University, Taichung, Taiwan

**Keywords:** phthalates, endocrine-disrupting chemicals, pubertal development, development of reproductive organs, birth cohort

## Abstract

**Purpose:**

Phthalates are ubiquitous endocrine disruptors that can affect pubertal development in children. The association of fetal and childhood levels of phthalates with pubertal development were explored.

**Methods:**

We conduct a population-based birth cohort study to investigate the association between prenatal and childhood exposure to phthalates and pubertal development. Initially, a total of 445 children were recruited from 2000 to 2001, of which 90 children were followed for 15 years which measurements of urine and development assessed at 2, 5, 8, 11, and 14 years. We defined higher Tanner stage as the 14-year-old Tanner stage ≥ 4 and 5 for boys and girls, respectively. A logistic regression analysis was conducted to estimate the crude and adjusted odds ratio of a higher Tanner stage at 14 years old. The Pearson correlation coefficient and multiple linear regression were used to estimate the association of testicular volume, uterine volume, ovarian volume, and blood hormones at 14 years of age with the log-transformed concentration of phthalates at 2, 5, 8, 11, and 14 years.

**Results:**

In boys, a significantly different geometric mean of mono-benzyl phthalate (MBzP) was observed in 11-year-olds; 6.82 and 2.96 in the lower Tanner stage group and higher Tanner stage group. In girls, a significant difference in the geometric mean of mono(2-ethyl-5-hydroxyhexyl) phthalate (MEHHP) in 11-year-olds and mono-ethyl phthalate (MEP) in 2-year-olds was observed; MEHHP was 32.97 and 18.13 in the lower Tanner stage group and higher Tanner stage group, and MEP was 26.54 and 65.74 in the lower Tanner stage group and higher Tanner stage group, respectively. Uterine volume at 14 years old was negatively associated with several phthalate metabolites (MEHP at 8 years old, MnBP at 8 years old, MBzP at 14 years old, MMP prenatally, MMP at 8 years old, and MEP at 8 years old) after adjusting for covariates. However, no significant correlations were found between phthalate metabolites and ovarian or testicular volume.

**Conclusion:**

Phthalate exposure at certain time points may influence the reproductive development of children during puberty; however, further studies should be conducted to determine the causal nature of this association.

## 1 Introduction

Phthalates, which are endocrine-disrupting chemicals (EDCs), are mainly used in plastic products as plasticizers, making them elastic and pliable. Humans are exposed to phthalates from a wide range of sources through inhalation, digestion, or dermal absorption (1, 2), including diet, unique hygiene products, cosmetics and skin products, medical applications, and recreational and pharmaceutical agents (2–5). Experimental and observational studies have suggested that phthalates can negatively affect male and female reproductive health *via* interference with estrogen/androgen production and/or inflammatory/oxidative stress pathways (6–11).

Prenatal, postnatal, and childhood exposure to phthalates affects hormone levels and pubertal development; however, the underlying mechanisms remain unclear (1, 12–15). Eales et al. (14) summarized the high-to-moderate health effects of phthalates and their metabolites, which include lower semen quality, impaired neurodevelopment, risk of childhood asthma, anogenital distance in boys, low birth weight, endometriosis, decreased testosterone levels, type 2 diabetes, and breast/uterine cancer. Basso et al. (15) considered that phthalates and their metabolites are crucial environmental factors contributing to the etiology of uterine and ovarian diseases in women, such as polycystic ovary syndrome and endometriosis.

Phthalates induce various cellular responses, including the modulation of the expression of steroid hormone receptors and transcription and paracrine factors. For example, male rats treated with di(2-ethylhexyl)phthalate (DEHP) exhibited delayed puberty and changes in serum anti-Mullerian hormone (AMH), estrogen levels, follicle-stimulating hormone (FSH), and gene expression (16). The research findings indicate that exposure to DEHP in animal experiments is linked to reduced gene expression and expression of critical proteins. This exposure may contribute to the development of defects, such as abnormal uterine structure, epithelial proliferation, uterine receptivity, and endometrial shedding in rats (17, 18). These results highlight the potential adverse effects of DEHP exposure on reproductive health and provide insight into the mechanisms by which DEHP affects the reproductive system. Further studies are needed to investigate the long-term effects of DEHP exposure and its impact on human health.

The age of puberty onset varies widely among individuals across ethnicities and sexes (6–10, 19). Puberty begins between the ages of 8 and 12 years in girls and 9 and 14 years in boys (20–22). The exact mechanism that triggers puberty onset remains elusive (20–24). Kisspeptins belonging to the neuropeptide family may be associated with female puberty initiation and ovulation regulation through the central control of the hypothalamus–pituitary–gonad axis. Increased prenatal or early childhood exposure to phthalates positively correlates with elevated kisspeptin concentrations, suggesting that such exposure may accelerate sexual maturation in girls (22, 25–27). Although epidemiological studies have limitations, they suggest that phthalates may affect reproductive outcomes and children’s health.

The human development cycle is classified into infants, toddlers, preschool- and school-aged children, adolescents, and young adults, before reaching adulthood (28). Our previous studies on phthalate metabolites have indicated that the phthalate exposure concentration might affect sex steroid hormone levels and negatively affect uterus development in girls (7, 8). Most cohort epidemiological studies have focused on the effects of gestational exposure to maternal or neonatal hormones on childhood puberty development or adulthood reproductive health; however, sensitivity to phthalate exposure is not limited to the fetal and neonatal periods. In this study, we examined the association between prenatal to childhood phthalate exposure (prenatally and at 2, 5, 8, and 11 years old) and the development of puberty and reproductive organs in 14-year-old children.

## 2 Methods

**Figure 1:**
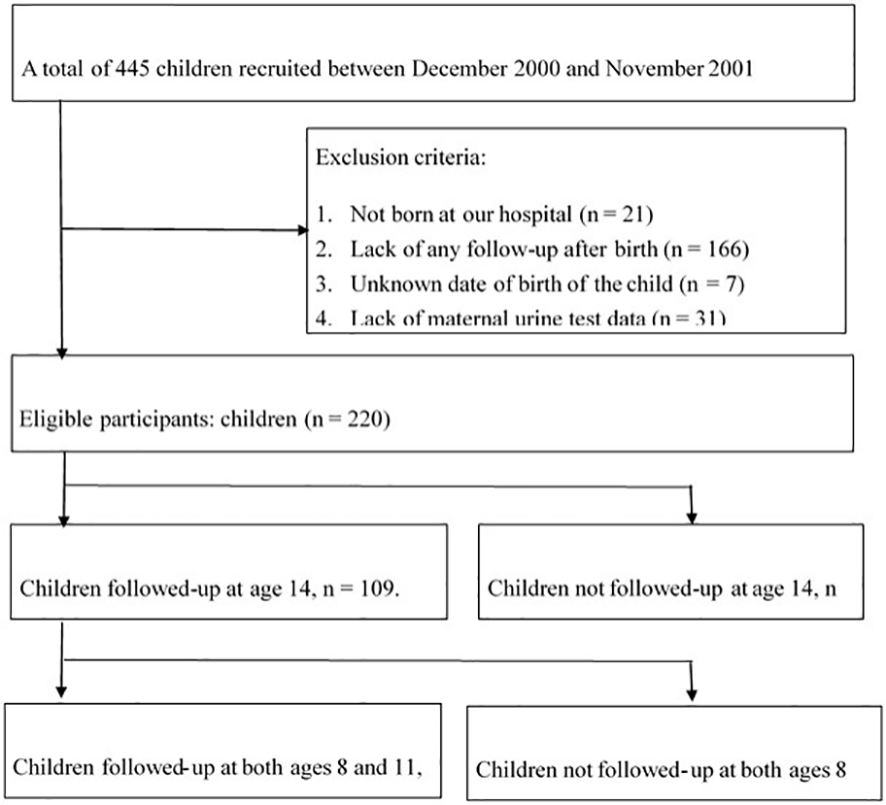
Flowchart of the recruitment of women and follow-up of children. During the follow-up period, phthalate metabolites in the urine from mothers and children were measured at 2, 5, 8, 11, and 14 years of age.

This study conducted a 15-year follow-up of the effect of prenatal and childhood exposure to phthalates on pubertal development in a population-based birth cohort. Initially, a total of 445 children were recruited from 2000 to 2001. Twenty-one children were excluded who were not born in our hospital, 166 children who had never attended follow-up since birth, seven children whose birth dates were unknown, and 31 children without a maternal urine test data. Among the 220 eligible children, a total of 90 completed all three surveys at 8, 11, and 14 years of age during the follow-up period, while the remaining 130 children did not provide complete responses. Thus, the overall response rate for the study was 40.9% (90/220). **Figure 1** illustrates a flowchart describing the details of the study population.

This study was approved by the Ethics Review Committee of the National Health Research Institutes in Taiwan, and informed written consent was obtained before the commencement of the study from each of the pregnant participants, the guardian, and the children ≥6 years of age using suitable language for children’s understanding.

### 2.1 Phthalate metabolite and urine creatinine measurements

The researchers collected one-spot maternal urine samples from women in their third trimester (28–36 weeks of gestation). Urine from children was collected independently at ages 2, 5, 8, 11, and 14 years. Urine samples were collected using standard collection containers at the hospital and stored at 4°C until transportation to the analytical laboratory at the National Health Research Institutes (NHRI) by trained research personnel. Upon arrival at the laboratory, the samples were immediately aliquoted and frozen at -20°C to ensure preservation and enable future analysis. All phthalate metabolites were tested and strictly controlled by the NHRI within a period of 2 weeks.

Maternal and children phthalate exposure was assessed using the urinary concentration of seven phthalate metabolites, including mono(2-ethylhexyl) phthalate (MEHP), mono(2-ethyl-5-hydroxyhexyl) phthalate (MEHHP), mono(2-ethyl-5-oxohexyl) phthalate (MEOHP), mono-n-butyl phthalate (MnBP), mono-benzyl phthalate (MBzP), mono-methyl phthalate (MMP), and mono-ethyl phthalate (MEP). Total DEHP levels were determined as the sum of MEHP, MEHHP, and MEOHP levels. The National Health Research Institutes (NHRI) analytical laboratory used an online solid phase extraction system coupled with liquid chromatography electrospray ionization tandem mass spectrometry (LC–ESI–MS/MS), which was validated by an international laboratory comparison program (G-EQUAS 59, the German External Quality Assessment Scheme, Erlangen, Germany). As part of the pretreatment process, the ^13^C_4_ isotopes of the phthalate metabolites were added as internal standards to the urine samples prior to deconjugation using β-glucuronidase. This was done to facilitate accurate quantification of the target analytes by correcting for variations in sample preparation, matrix effects, and instrument drift. Metabolite concentrations adjusted for creatinine were denoted in micrograms per grams of creatinine (µg/gC). Urinary creatinine levels were determined at Kaohsiung Medical University Chung-Ho Memorial Hospital using spectrophotometric methods, with picric acid as the reactive agent and the reader set at 520 nm.

### 2.2 Sex hormone measurements

Serum total testosterone (TT) and progesterone levels were measured using a solid-phase, competitive chemiluminescent enzyme immunoassay (Immulite 2000 Systems Analyzers, Siemens Medical Solutions Diagnostics, Deerfield, Illinois). The sensitivity for TT was 1.5 ng/mL, and for progesterone was 0.1 ng/mL (0.3 nmol/L). Serum estradiol (E2) levels were measured using a radioimmunoassay kit (Diagnostic Systems Laboratories, Santa Monica, California), and the sensitivity of this assay was 0.22 ng/dL. Serum follicle-stimulating (FSH) and luteinizing hormone (LH) levels were measured *via* enzyme immunoassays (FSH: Abbott Laboratories, Rome, Italy; LH: Dade Behring, Milan, Italy). The sensitivity for both assays was 0.2mIU/ml. Intra- and interassay coefficients of variations were 4.7% and 8.9%, respectively, for FSH, and 3.1% and 4.0%, respectively, for LH. The sex hormone binding globulin (SHBG) levels were measured using a solid-phase “sandwich” immunoradiometric assay comprising two monoclonal antibodies directed against two different antigen sites, one of which is radiolabeled with iodine125 (Cisbio Bioassays, Codolet, France). This radioimmunoassay had analytical and functional detection limits below 0.5 nmol/L, an intra-assay coefficient of variation (CV) of 5.2%, and inter-assay CVs of 5.3%.

### 2.3 Evaluation of pubertal development at 8, 11, and 14 years of age

Successive physical examinations and clinical assessments of reproductive development were conducted by a pediatric specialist (P-H Su) every 2–3 years. The recorded pubertal landmarks of the children aged 8, 11, and 14 years were used in the present study. According to the established methodology of Greulich and Pyle (29), we calculated bone age (BA) using radiographs of the left hand and determined the ratio of BA to chronological age (BA/CA). Testicular volume was assessed by comparison with a reference orchidometer, which is a standard tool for evaluating testicular size in a consistent and accurate manner (30).

Complete pelvic organ examination of all girls was conducted through full-bladder transabdominal ultrasound with a transabdominal probe set at 3.64 MHz (Acuson Antares Ultrasound system, Siemens Medical Solutions, Malvern, PA, USA) (31). Uterine measurements included the longitudinal diameter, transverse diameter, uterine body length, and cervical length. Bilateral ovarian size measurements included the longitudinal and transverse diameters. A senior specialist blinded to exposure levels performed ultrasound scans using a standard protocol. The reliability of the ultrasound measurements for uterine size and ovarian volume was tested, with the average CVs ranging from 2.82% to 8.76%. In a single assessment session, six participants were scanned five times each due to the inability to verify the obtained results. CVs were < 15%.

The development of genital and armpit hair was measured according to the patterns of pubertal change defined by Tanner (32). The highest stage among these signs was recorded for each child by sex at the ages of 8, 11, and 14 years. The Tanner stages include five stages, ranging from stage 1 (pre-pubertal) to stage 5 (fully mature). A brief overview of the Tanner stages is as follows:

Stage 1: Pre-pubertal, no development of secondary sexual characteristics.
Stage 2: Initial development of secondary sexual characteristics; for boys, testicular enlargement and fine pubic hair, and for girls, breast budding and fine pubic hair.
Stage 3: Further development of secondary sexual characteristics; for boys, penis growth and pubic hair darkening, and for girls, continued breast growth and pubic hair darkening.
Stage 4: Advanced development of secondary sexual characteristics; for boys, increased penis and testicular size and more adult-like pubic hair, and for girls, the breast assumes an adult shape, and pubic hair takes an adult pattern.
Stage 5: Full maturity; for boys, adult genitalia and pubic hair, and for girls, adult breasts and pubic hair.

### 2.4 Statistical analysis

All demographics and sexual characteristics are presented as mean ± standard deviation (SD) for continuous data and as numbers and percentages (n, %) for categorical data. The phthalate data are presented as geometric means [95% confidence interval (CI)] and were compared between Tanner stage < 4 and Tanner stage ≥ 4 in boys and Tanner stage < 5 and Tanner stage = 5 in girls using the Student’s *t*-test for log-normally distributed data. The geometric means of phthalate levels at six follow-up time points (prenatally and at 2, 5, 8, 11, and 14 years old) were compared using a two-sample t-test. A logistic regression analysis was conducted to estimate the crude and adjusted odds ratio (aOR) of puberty development (Tanner stage ≥ 4 and 5 for boys and girls, respectively), which yielded the log-linear association with the log-transformed concentration of phthalates at six-time points. The Pearson correlation coefficient was estimated for the association of the testicular volume, uterine volume, ovarian volume, and blood hormones at 14 years of age with the log-transformed concentration of phthalates at each follow-up time point. Linear regression was conducted to determine the adjusted association of the testicular volume, uterine volume, ovarian volume, and blood hormones with the log-transformed concentration of phthalates at each follow-up time point. All statistical assessments were two-tailed and considered significant at p < 0.05. All data were analyzed using SAS version 9.4 (SAS Institute, Cary, NC, USA).

## 3 Results

### 3.1 Characteristics of the study population

The maternal and demographic characteristics of children who were followed up to 14 years of age (n = 90) and those lost to follow-up (n = 130) are presented in **Supplementary Table S1**. The distribution of maternal characteristics and phthalate metabolite levels during pregnancy was not significantly different between the two follow-up groups.

### 3.2 Characteristics of pubertal development at 14 years of age

**Figure 2:**
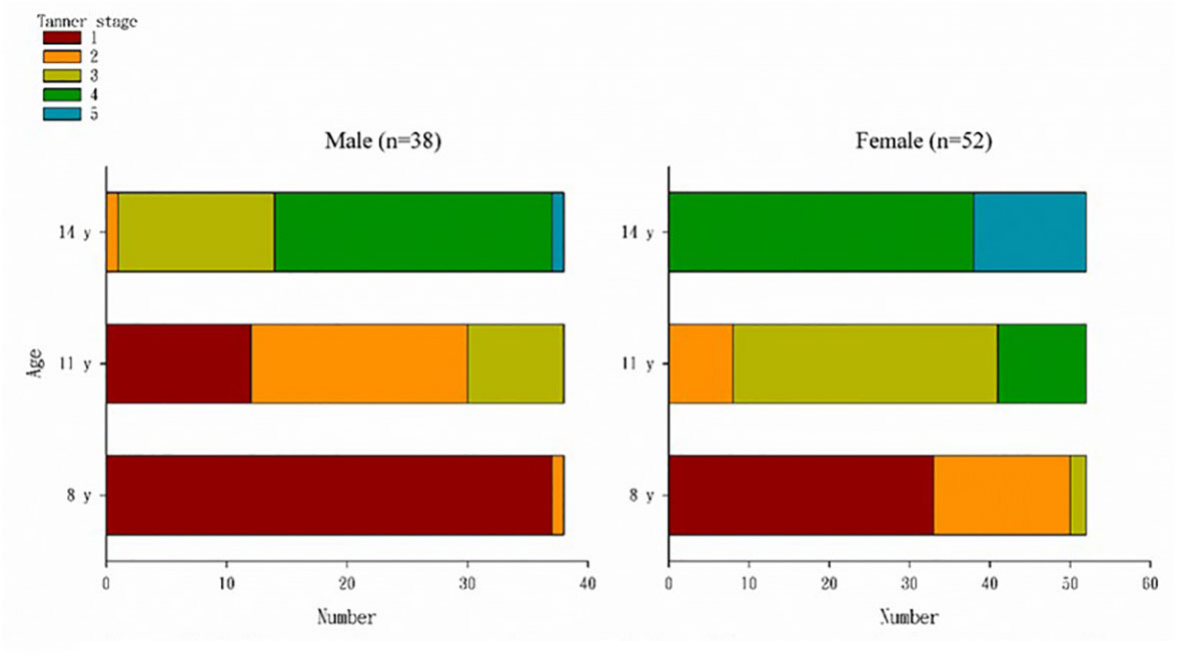
Distribution of Tanner stages at the ages of 8, 11, and 14 years by sex. At the age of 8 years, 37 (97%) boys were in Tanner stage 1, 33 (63.5%) girls were in Tanner stage 1, 17 (32.7%) were in Tanner stage 2, and two (3.9%) were Tanner stage 3. At the age of 11 years, 12 boys (31.6%) were still in Tanner stage 1, eight (21.1%) were in Tanner stage 3, and among girls, 33 (63.5%) were in Tanner stage 3, and 11 (21.2%) were in Tanner stage 3 and had entered the fourth stage of Tanner. At the age of 14 years, one boy was still in Tanner stage 1, and most had entered Tanner stage 3 (13, 34.2%) and 4 (23, 60.5%); 38 (73.1%) girls had entered Tanner stage 4, and 14 (26.9%) were in Tanner stage 5.

The distribution of Tanner stages at 8, 11, and 14 years of age among boys and girls is presented in **Figure 2**. **Table 1** presents the children’s demographic, body composition, genital, and blood hormone characteristics at 14 years of age stratified by sex and Tanner stage. Overall, 14 boys (36.8%) were Tanner stage < 4, and 38 girls (73.1%) were Tanner stage < 5. For boys, the BA (13.61 ± 0.40) of Tanner stage < 4 was significantly (p < 0.0001) lower than that of Tanner stage ≥ 4 (14.85 ± 0.51). For girls, the BA (13.95 ± 0.35) of Tanner stage < 5 was significantly (p < 0.0001) lower than that of Tanner stage 5 (14.71 ± 0.43). The weight (50.24 ± 11.43 kg), body mass index (BMI; 18.49 ± 2.63), and testicular volume (15.57 ± 3.98) of boys with Tanner stage < 4 were significantly lower than those of boys with Tanner stage ≥ 4 (60.00 ± 11.45, p = 0.0157; 21.09 ± 3.70, p = 0.0272; and 19.92 ± 2.50, p = 0.0002, respectively). The weight (52.24 ± 9.98 kg) and BMI (20.29 ± 3.29) of girls with Tanner stage < 5 were lower than those of girls with Tanner stage 5 (62.57 ± 15.83, p = 0.0358 and 24.27 ± 5.00, p = 0.0130, respectively), but the differences in female uterus and ovary size were non-significant. For boys, no significant difference was observed in blood hormones, including estradiol, TT, progesterone, FSH, LH, and SHBG, between the Tanner stage < 4 group and Tanner stage ≥ 4 group. For girls, we observed higher TT and progesterone levels and lower FSH levels in those with Tanner stage 5.

**Table 1:** Demographic, body composition, genital, and blood hormone characteristics in children aged 14 years stratified by sex and Tanner stage (n = 90).

Variables	Boys		p	Girls		p
	Tanner stage < 4	Tanner stage ≥ 4		Tanner stage < 5	Tanner stage = 5	
	n = 14	n = 24		n = 38	n = 14	
Demographic						
Maternal age at delivery†	29.64 ± 2.84	30.46 ± 3.58	0.4712	30.16 ± 3.69	31.29 ± 4.56	0.3636
Maternal education						
< 12 years	11 (78.6%)	19 (79.2%)	0.9654	32 (84.2%)	10 (71.4%)	0.2996
≧12 years	3 (21.4%)	5 (20.8%)		6 (15.8%)	4 (28.6%)	
Paternal education						
< 12 years	8 (57.1%)	16 (66.7%)	0.5571	31 (81.6%)	13 (92.9%)	0.3174
≧12 years	6 (42.9%)	8 (33.3%)		7 (18.4%)	1 (7.1%)	
Family income per year						
< 600,000 NTDs	6 (42.9%)	8 (33.3%)	0.5571	12 (32.4%)	4 (28.6%)	0.7909
≧600,000 NTDs	8 (57.1%)	16 (66.7%)		25 (67.6%)	10 (71.4%)	
Children Age, year†	13.79 ± 0.43	13.83 ± 0.38	0.7238	13.82 ± 0.39	13.79 ± 0.43	0.8117
Body composition†						
Bone age (year)	**13.61 ± 0.40**	**14.85 ± 0.51**	**<0.0001**	**13.95 ± 0.35**	**14.71 ± 0.43**	**<0.0001**
Height, cm	**163.67 ± 8.41**	**168.50 ± 5.43**	**0.0379**	160.17 ± 4.58	159.86 ± 5.38	0.8352
Weight (kg)	**50.24 ± 11.43**	**60.00 ± 11.45**	**0.0157**	**52.24 ± 9.98**	**62.57 ± 15.83**	**0.0358**
BMI (kg/cm^2^)	**18.49 ± 2.63**	**21.09 ± 3.70**	**0.0272**	**20.29 ± 3.29**	**24.27 ± 5.00**	**0.0130**
Genital†						
Testicular volume	**15.57 ± 3.98**	**19.92 ± 2.50**	**0.0002**			
Uterine volume				14.75 ± 5.33	14.92 ± 5.07	0.9154
Ovarian volume				5.07 ± 1.67	5.35 ± 1.85	0.6122
Blood hormone‡						
Estradiol (pg/mL)	35.00 (30.48-40.20)	40.26 (34.46-47.03)	0.2120	77.20 (61.90-96.29)	114.40 (70.04-186.90)	0.0857
Total testosterone (ng/mL)	531.80(456.00-620.20)	590.80(535.00-652.30)	0.2127	**47.50 (39.87-56.60)**	**67.17 (49.48-91.17)**	**0.0400**
Progesterone (ng/dL)	0.33 (0.27-0.41)	0.42 (0.36-0.48)	0.0645	**0.57 (0.40-0.81)**	**2.02 (0.92-4.43)**	**0.0008**
FSH (mIU/mL)	3.20 (2.30-4.46)	3.66 (3.14-4.27)	0.3833	**3.66 (2.99-4.47)**	**2.32 (1.46-3.70)**	**0.0329**
LH (mIU/mL)	1.64 (1.25-2.17)	2.06 (1.66-2.55)	0.1918	2.70 (2.00-3.65)	3.67 (2.39-5.63)	0.2616
SHBG (nmol/L)	28.48 (20.37-39.83)	21.75 (18.05-26.21)	0.1150	31.90 (27.49-37.01)	26.76 (17.54-40.82)	0.4122

†Values are presented as mean ± standard deviation and compared using independent Student’s t test.

‡Values are presented as geometric mean [95% confidence interval (95% CI)] and compared using Student’s t test for log-normally distributed samples.

FSH, Follicle-stimulating hormone; LH, Luteinizing hormone; SHBG, Sex hormone-binding globulin.

The bold values indicate significant results (p<0.05).

### 3.3 Phthalates and pubertal development

The association between different phthalate metabolites and pubertal development in children is presented in **Table 2**. The results indicated no associations between maternal phthalate metabolite concentrations and pubertal development in both male and female children at 14 years of age. In boys, a significant association was noted between the Tanner stage at 14 years of age and the geometric mean of MBzP (μg/g creatinine) at 11 years old (6.82 in Tanner stage < 4 vs. 2.96 in Tanner stage ≥ 4, p = 0.0390). In girls, a significant difference was observed in the geometric mean of MEHHP (μg/g creatinine) at 11 years old (32.97 in Tanner stage < 5 vs. 18.13 in Tanner stage 5, p = 0.0497) and MEP (μg/g creatinine) at 2 years old (26.54 in Tanner stage < 5 vs. 65.74 in Tanner stage 5, p = 0.0420). **Table 3** presents the findings of the logistic regression analysis. After adjustment for the covariates, including maternal age at delivery, maternal education, paternal education, and family income, the aOR of the higher Tanner stage (≥ 4 for boys and = 5 for girls) at 14 years of age for exposure to urinary phthalate metabolites were estimated. For boys, MBzP at 11 years old was negatively associated with a higher Tanner stage at 14 years of age; the aOR was 0.450 (95% CI: 0.204–0.993) per one increment of log-transformed MBzP (μg/g creatinine). For girls, MEHP at 11 years old (aOR = 0.404, 95% CI: 0.182–0.898) and MEHHP at 11 years old (aOR = 0.158, 95% CI: 0.038–0.653) were negatively associated with a higher Tanner stage at 14 years of age; however, MEP at 2 years old (aOR = 7.201, 95% CI: 1.098–47.221) was positively associated with a higher Tanner stage at 14 years of age.

**Table 2:** Geometric means (95% confidence intervals) of urinary phthalate metabolite levels (μg/g creatinine) prenatally and at 2, 5, 8, 11, and 14 years of age stratified by Tanner stage at 14 years of age.

	Boys			Girls		
	Tanner stage < 4	Tanner stage ≥ 4	p	Tanner stage < 5	Tanner stage = 5	p
	n = 14	n = 24		n = 38	n = 14	
MEHP						
At prenatal	16.78(10.47-26.88)	21.62(13.56-34.45)	0.4248	18.56(13.28-25.94)	15.80(11.48-21.75)	0.4722
At 2 years old	20.10(12.03-33.57)	17.97(11.44-28.22)	0.7215	22.05(15.99-30.40)	17.04(7.26-39.97)	0.5263
At 5 years old	14.60(7.89-27.01)	14.34(8.22-25.04)	0.9639	8.99(5.01-16.13)	15.35(6.45-36.53)	0.2797
At 8 years old	8.65(5.32-14.06)	9.17(5.63-14.94)	0.8575	8.58(5.50-13.40)	7.43(3.06-18.05)	0.7583
At 11 years old	12.93(7.47-22.39)	10.99(7.71-15.66)	0.6000	9.04(6.71-12.18)	5.25(2.80-9.86)	0.1120
At 14 years old	5.02(3.29-7.67)	4.63(3.13-6.87)	0.7699	2.85(1.87-4.36)	1.98(0.86-4.55)	0.4146
MEHHP						
At prenatal	6.27(2.13-18.48)	17.81(9.12-34.77)	0.0927	4.76(2.34-9.66)	4.89(1.72-13.86)	0.9647
At 2 years old	76.74(42.25-139.39)	132.56(86.63-202.86)	0.1153	95.35(68.43-132.85)	119.76(38.25-374.91)	0.6650
At 5 years old	97.81(55.96-170.95)	67.68(45.52-100.62)	0.2565	75.13(48.94-115.35)	124.93(58.68-265.97)	0.2178
At 8 years old	40.94(28.91-57.97)	43.29(28.88-64.89)	0.8266	44.19(33.56-58.19)	37.17(22.20-62.22)	0.5323
At 11 years old	43.55(28.96-65.50)	32.01(24.56-41.72)	0.1895	**32.97(27.29-39.83)**	**18.13(10.19-32.23)**	**0.0497**
At 14 years old	16.83(11.83-23.93)	13.87(10.91-17.62)	0.3422	14.80(12.45-17.58)	11.44(8.56-15.28)	0.1181
MEOHP						
At prenatal	9.43(4.22-21.07)	21.25(10.18-44.35)	0.1241	11.99(7.37-19.51)	15.12(11.70-19.54)	0.3917
At 2 years old	59.86(32.17-111.37)	69.96(32.79-149.30)	0.7313	67.52(51.10-89.22)	75.58(27.73-206.04)	0.8059
At 5 years old	62.37(43.07-90.32)	44.90(33.07-60.94)	0.1518	41.63(27.30-63.49)	66.62(35.36-125.53)	0.1944
At 8 years old	35.73(26.03-49.04)	38.16(25.64-56.79)	0.7867	36.85(28.01-48.49)	31.58(17.81-56.01)	0.6082
At 11 years old	25.15(10.72-58.96)	27.42(21.40-35.13)	0.8365	20.12(13.54-29.91)	9.68(2.97-31.56)	0.2254
At 14 years old	12.53(9.13-17.20)	9.32(7.10-12.23)	0.1431	9.40(7.51-11.77)	7.37(5.31-10.24)	0.2083
ΣDEHP						
At prenatal	41.24(24.29-70.03)	76.24(45.64-127.37)	0.0872	46.93(33.64-65.48)	41.89(32.81-53.48)	0.5713
At 2 years old	160.62(90.65-284.58)	241.12(160.16-363.00)	0.2163	193.68(146.53-256.01)	217.49(75.46-626.87)	0.8101
At 5 years old	183.54(114.50-294.21)	137.75(97.72-194.17)	0.2981	134.05(89.75-200.23)	227.71(119.19-435.02)	0.1461
At 8 years old	86.86(62.39-120.94)	94.16(63.48-139.67)	0.7433	96.22(70.88-130.63)	79.65(45.10-140.67)	0.5375
At 11 years old	89.58(57.79-138.85)	71.88(55.29-93.46)	0.3673	67.73(55.71-82.35)	38.90(21.80-69.44)	0.0690
At 14 years old	35.24(25.30-49.09)	28.39(21.92-36.78)	0.2840	29.05(24.45-34.53)	21.75(15.55-30.42)	0.1166
MnBP						
At prenatal	64.49(41.38-100.52)	76.95(53.61-110.44)	0.5177	63.36(44.92-89.38)	91.26(51.41-162.01)	0.2583
At 2 years old	196.64(106.29-363.77)	213.02(137.64-329.68)	0.8167	150.69(116.00-195.76)	180.74(60.77-537.51)	0.7134
At 5 years old	189.06(76.25-468.75)	166.85(89.25-311.91)	0.8083	94.43(78.83-113.12)	127.98(83.01-197.31)	0.1754
At 8 years old	78.17(53.30-114.64)	77.24(54.89-108.68)	0.9609	121.43(94.46-156.09)	93.04(55.20-156.80)	0.3360
At 11 years old	47.46(32.43-69.45)	48.34(38.69-60.41)	0.9297	46.51(38.74-55.84)	47.62(25.90-87.56)	0.9373
At 14 years old	17.75(12.65-24.90)	26.11(18.75-36.37)	0.0942	22.83(18.67-27.91)	22.54(14.71-34.53)	0.9548
MBzP						
At prenatal	12.18(9.02-16.44)	14.29(9.17-22.29)	0.5353	17.82(13.66-23.24)	10.99(6.22-19.43)	0.1169
At 2 years old	11.87(5.70-24.70)	5.49(2.24-13.45)	0.1593	8.74(5.31-14.37)	4.58(2.30-9.13)	0.1056
At 5 years old	19.94(11.22-35.44)	22.24(13.69-36.14)	0.7560	11.61(8.28-16.28)	16.75(11.34-24.75)	0.1380
At 8 years old	10.11(5.45-18.78)	10.78(5.85-19.87)	0.8777	12.00(8.22-17.53)	16.77(4.57-61.51)	0.6004
At 11 years old	**6.82(3.34-13.90)**	**2.96(2.01-4.36)**	**0.0390**	2.99(2.07-4.31)	2.32(1.27-4.24)	0.4558
At 14 years old	0.84(0.36-1.96)	0.51(0.26-1.03)	0.3439	0.72(0.39-1.35)	1.56(0.53-4.62)	0.2047
MMP						
At prenatal	74.37(41.98-131.75)	39.60(24.17-64.88)	0.0869	46.02(31.47-67.30)	73.29(44.37-121.06)	0.1293
At 2 years old	14.02(7.70-25.54)	25.54(17.16-38.03)	0.0813	15.48(11.95-20.03)	14.80(9.76-22.45)	0.8390
At 5 years old	16.13(7.37-35.29)	25.17(10.90-58.11)	0.4113	15.96(11.81-21.58)	21.60(12.07-38.65)	0.3268
At 8 years old	8.85(5.82-13.46)	6.22(3.61-10.70)	0.2865	8.87(6.43-12.24)	7.05(3.31-15.02)	0.5542
At 11 years old	7.67(2.55-23.12)	8.24(4.37-15.56)	0.9054	10.58(6.86-16.32)	10.31(5.88-18.10)	0.9396
At 14 years old	1.99(0.64-6.18)	2.33(1.00-5.39)	0.8132	2.67(1.34-5.33)	1.40(0.38-5.17)	0.3614
MEP						
At prenatal	77.32(38.28-156.20)	55.01(41.21-73.43)	0.3492	60.73(44.46-82.95)	60.37(39.40-92.50)	0.9814
At 2 years old	28.79(9.46-87.61)	26.22(19.53-35.19)	0.8582	**26.54(18.12-38.86)**	**65.74(28.76-150.29)**	**0.0420**
At 5 years old	28.11(11.43-69.10)	20.78(12.29-35.12)	0.5356	14.94(9.56-23.34)	26.71(16.10-44.30)	0.0752
At 8 years old	11.98(6.33-22.69)	13.82(9.10-21.00)	0.6935	19.32(12.75-29.26)	15.97(9.99-25.54)	0.5269
At 11 years old	17.32(6.43-46.64)	11.83(5.13-27.25)	0.5366	5.19(2.65-10.15)	12.99(5.22-32.34)	0.0976
At 14 years old	6.30(2.16-18.33)	7.24(3.15-16.63)	0.8279	1.94(0.86-4.42)	3.88(0.89-16.87)	0.3915

p value was estimated using Student’s t test for log-normally distributed samples

The bold values indicate significant results (p<0.05).

**Table 3:** Logistic regression analysis findings showing the relationship between higher Tanner stage (≥ 4 for boys, and 5 for girls) at 14 years of age and log-transformed values of urinary phthalate metabolite levels (μg/g creatinine) prenatally and at 2, 5, 8, 11, and 14 years of age.

Log-transformed value of urinary phthalate metabolite	Boys		Girls	
	Crude OR (95% CI)	Adjusted OR (95% CI)	Crude OR(95% CI)	Adjusted OR (95% CI)
MEHP				
At prenatal	1.318(0.641-2.710)	1.166(0.544-2.499)	0.817(0.406-1.645)	0.746(0.340-1.637)
At 2 years old	0.825(0.290-2.351)	0.877(0.242-3.180)	0.669(0.243-1.842)	0.850(0.289-2.501)
At 5 years old	0.984(0.496-1.954)	1.146(0.546-2.403)	1.329(0.778-2.271)	0.893(0.418-1.908)
At 8 years old	1.058(0.556-2.014)	1.065(0.532-2.133)	0.922(0.569-1.494)	0.843(0.505-1.409)
At 11 years old	0.804(0.374-1.728)	0.891(0.405-1.960)	0.556(0.287-1.077)	**0.404(0.182-0.898)**
At 14 years old	0.891(0.402-1.976)	1.046(0.433-2.525)	0.815(0.517-1.285)	0.797(0.480-1.323)
MEHHP				
At prenatal	1.457(0.944-2.249)	1.447(0.930-2.250)	1.006(0.744-1.362)	1.018(0.732-1.417)
At 2 years old	2.450(0.802-7.482)	3.292(0.830-13.063)	1.300(0.550-3.074)	1.639(0.600-4.483)
At 5 years old	0.590(0.244-1.428)	0.525(0.199-1.381)	1.523(0.792-2.929)	1.511(0.616-3.704)
At 8 years old	1.087(0.484-2.438)	1.208(0.519-2.812)	0.754(0.320-1.775)	0.589(0.242-1.433)
At 11 years old	0.487(0.171-1.386)	0.505(0.174-1.462)	**0.311(0.113-0.856)**	**0.158(0.038-0.653)**
At 14 years old	0.556(0.174-1.782)	0.638(0.180-2.261)	0.349(0.092-1.329)	0.255(0.054-1.198)
MEOHP				
At prenatal	1.399(0.884-2.215)	1.333(0.832-2.135)	1.161(0.700-1.926)	1.199(0.656-2.193)
At 2 years old	1.113(0.580-2.132)	1.483(0.663-3.315)	1.194(0.439-3.247)	1.462(0.489-4.377)
At 5 years old	0.408(0.115-1.448)	0.343(0.088-1.343)	1.615(0.758-3.443)	1.561(0.543-4.491)
At 8 years old	1.110(0.481-2.561)	1.228(0.515-2.928)	0.793(0.353-1.782)	0.619(0.267-1.438)
At 11 years old	1.093(0.562-2.126)	1.213(0.598-2.462)	0.745(0.505-1.100)	0.607(0.357-1.032)
At 14 years old	0.430(0.132-1.404)	0.456(0.122-1.712)	0.569(0.218-1.485)	0.526(0.181-1.531)
ΣDEHP				
At prenatal	1.806(0.854-3.821)	1.649(0.778-3.499)	0.863(0.429-1.738)	0.842(0.382-1.856)
At 2 years old	2.038(0.676-6.142)	3.023(0.759-12.047)	1.188(0.449-3.146)	1.495(0.506-4.418)
At 5 years old	0.572(0.205-1.594)	0.542(0.179-1.643)	1.700(0.812-3.559)	1.475(0.556-3.917)
At 8 years old	1.136(0.492-2.626)	1.228(0.518-2.909)	0.780(0.363-1.676)	0.636(0.285-1.418)
At 11 years old	0.611(0.225-1.657)	0.671(0.241-1.870)	**0.361(0.142-0.919)**	**0.200(0.054-0.739)**
At 14 years old	0.531(0.167-1.694)	0.603(0.166-2.193)	0.342(0.096-1.220)	0.282(0.068-1.166)
MnBP				
At prenatal	1.314(0.574-3.005)	1.293(0.530-3.155)	1.422(0.770-2.627)	1.237(0.629-2.434)
At 2 years old	1.131(0.424-3.017)	1.458(0.464-4.579)	1.311(0.495-3.472)	1.563(0.520-4.703)
At 5 years old	0.931(0.539-1.605)	0.838(0.462-1.518)	3.330(0.741-14.961)	3.128(0.576-16.999)
At 8 years old	0.978(0.402-2.384)	0.858(0.321-2.291)	0.626(0.260-1.507)	0.509(0.195-1.326)
At 11 years old	1.060(0.329-3.413)	1.067(0.310-3.668)	1.049(0.438-2.512)	0.966(0.382-2.446)
At 14 years old	2.268(0.786-6.542)	2.305(0.777-6.838)	0.969(0.369-2.545)	0.733(0.253-2.122)
MBzP				
At prenatal	1.224(0.581-2.578)	1.221(0.563-2.649)	0.516(0.241-1.105)	0.427(0.174-1.048)
At 2 years old	0.671(0.362-1.244)	0.744(0.377-1.467)	0.554(0.242-1.267)	0.507(0.165-1.556)
At 5 years old	1.139(0.511-2.540)	1.449(0.533-3.937)	1.814(0.727-4.527)	1.396(0.523-3.725)
At 8 years old	1.039(0.621-1.740)	1.078(0.616-1.886)	1.164(0.768-1.766)	1.122(0.721-1.747)
At 11 years old	**0.450(0.214-0.945)**	**0.450(0.204-0.993)**	0.804(0.451-1.432)	0.774(0.387-1.551)
At 14 years old	0.807(0.516-1.264)	0.817(0.505-1.321)	1.245(0.890-1.743)	1.234(0.868-1.753)
MMP				
At prenatal	0.502(0.217-1.164)	0.394(0.145-1.075)	1.533(0.823-2.852)	1.661(0.800-3.450)
At 2 years old	3.436(0.835-14.129)	3.259(0.752-14.128)	0.865(0.202-3.702)	0.345(0.040-2.943)
At 5 years old	1.224(0.736-2.037)	1.211(0.710-2.065)	1.653(0.656-4.163)	1.308(0.453-3.776)
At 8 years old	0.740(0.395-1.387)	0.701(0.349-1.408)	0.808(0.440-1.483)	0.728(0.382-1.387)
At 11 years old	1.028(0.685-1.542)	0.970(0.595-1.579)	0.983(0.594-1.625)	1.035(0.592-1.809)
At 14 years old	1.043(0.744-1.463)	1.109(0.758-1.623)	0.869(0.654-1.154)	0.799(0.571-1.118)
MEP				
At prenatal	0.659(0.313-1.386)	0.580(0.251-1.340)	0.992(0.495-1.990)	0.847(0.401-1.787)
At 2 years old	0.913(0.419-1.989)	0.603(0.219-1.662)	**3.299(1.013-10.747)**	**7.201(1.098-47.221)**
At 5 years old	0.810(0.442-1.485)	0.642(0.318-1.295)	1.798(0.840-3.850)	1.519(0.672-3.435)
At 8 years old	1.156(0.587-2.275)	1.096(0.541-2.217)	0.861(0.489-1.519)	0.885(0.475-1.647)
At 11 years old	0.892(0.617-1.289)	0.835(0.565-1.234)	1.292(0.919-1.816)	1.261(0.858-1.854)
At 14 years old	1.040(0.734-1.473)	1.161(0.788-1.710)	1.121(0.870-1.445)	1.214(0.913-1.615)

OR, odds ratio. CI, confidence interval.

Adjusted OR, the odds ratio of higher Tanner stage (≥ 4 for boys and 5 for girls) at 14 years of age for urinary phthalate metabolite levels prenatally and at 2, 5, 8, 11, and 14 years of age was estimated with covariates including maternal age at delivery, maternal education, paternal education, and family income.

The bold values indicate significant results (p<0.05).

The Pearson correlations of the testicular volume, uterine volume, and ovarian volume at 14 years of age with log-transformed urinary phthalate metabolites prenatally and at 2, 5, 8, 11, and 14 years old are shown in **Supplementary Table S2**. The uterine volume had a significantly negative correlation with MEHP at 8 years old (r = −0.314), MEOHP at 2 years old (r = −0.367), MnBP at birth (r = −0.305), MnBP at 8 years old (r = −0.353), MBzP at 14 years old (r = −0.347), MMP at birth (r = −0.464), and MMP at 8 years old (r = −0.277). After adjustment for covariates, including maternal age at delivery, maternal education, paternal education, and family income, significant linear associations were found between the uterine volume at 14 years old and MEHP at 8 years old (slope = −1.155, R^2 = ^0.143), MnBP at 8 years old (slope = −2.382, R^2 = ^0.182), MBzP at 14 years old (slope = −0.906, R^2 = ^0.158), MMP prenatally (slope = −2.196, R^2 = ^0.242), MMP at 8 years old (slope = −1.434, R^2 = ^0.136), and MEP at 8 years old (slope = −1.342, R^2 = ^0.138) (**Table 4**).

**Table 4:** Multivariable linear model to estimate the effect of log-transformed urinary phthalate metabolites prenatally and at 2, 5, 8, 11, and 14 years old on puberty development at 14 years of age.

Log-transformed value of urinary phthalate metabolite	Independent variable: puberty development at 14 years old					
	Boys: Testicular volume (ml)		Girls: Uterine volume (cm^3^)		Girls: Ovarian volume (cm^3^)	
	Slope	R^2^	Slope	R^2^	Slope	R^2^
MEHP						
At prenatal	0.118	0.025	-1.266	0.097	-0.267	0.053
At 2 years old	-0.902	0.209	-1.106	0.104	0.235	0.083
At 5 years old	0.504	0.025	0.641	0.077	0.167	0.062
At 8 years old	-0.356	0.033	-1.155*	0.143	-1.342	0.138
At 11 years old	-0.411	0.032	0.292	0.054	0.496	0.081
At 14 years old	-0.735	0.047	-0.848	0.094	-0.211	0.061
MEHHP						
At prenatal	0.378	0.053	-0.062	0.052	-0.047	0.074
At 2 years old	-0.204	0.182	-1.642	0.149	-0.116	0.205
At 5 years old	0.318	0.011	-0.486	0.069	0.103	0.139
At 8 years old	0.385	0.031	-0.738	0.067	0.445	0.135
At 11 years old	-0.753	0.041	0.266	0.052	0.271	0.085
At 14 years old	-1.816	0.091	-1.256	0.065	0.471	0.090
MEOHP						
At prenatal	0.505	0.068	0.240	0.054	-0.245	0.105
At 2 years old	0.045	0.180	-2.605	0.202	-0.365	0.232
At 5 years old	0.321	0.008	-0.419	0.066	0.177	0.144
At 8 years old	0.221	0.026	-0.912	0.075	0.307	0.113
At 11 years old	-0.339	0.031	0.184	0.054	-0.050	0.073
At 14 years old	-2.259	0.131	-1.639	0.092	0.118	0.073
ΣDEHP						
At prenatal	0.308	0.032	1.054	0.081	0.188	0.080
At 2 years old	-0.292	0.183	-2.042	0.163	-0.230	0.214
At 5 years old	0.550	0.017	-0.367	0.065	0.056	0.136
At 8 years old	0.145	0.025	-1.138	0.093	0.253	0.108
At 11 years old	-0.767	0.042	0.372	0.054	0.188	0.078
At 14 years old	-1.912	0.099	-1.570	0.076	0.399	0.086
MnBP						
At prenatal	0.548	0.037	-1.424	0.119	-0.056	0.072
At 2 years old	0.608	0.196	-0.179	0.082	-0.381	0.235
At 5 years old	-0.551	0.043	0.822	0.067	-0.258	0.141
At 8 years old	0.133	0.024	-2.382*	0.182	-0.035	0.090
At 11 years old	1.869	0.103	1.011	0.068	-0.088	0.072
At 14 years old	-0.307	0.027	1.345	0.076	-0.237	0.078
MBzP						
At prenatal	-0.557	0.041	0.328	0.054	0.102	0.074
At 2 years old	0.281	0.190	0.372	0.085	0.272	0.226
At 5 years old	1.386	0.096	1.994	0.140	0.158	0.140
At 8 years old	0.213	0.029	-0.681	0.087	0.144	0.104
At 11 years old	-0.438	0.038	0.029	0.051	0.009	0.071
At 14 years old	-0.608	0.082	-0.906*	0.158	-0.023	0.072
MMP						
At prenatal	-0.503	0.047	-2.196**	0.242	0.054	0.072
At 2 years old	0.400	0.187	-0.232	0.082	-0.209	0.205
At 5 years old	0.181	0.011	-0.224	0.062	0.005	0.135
At 8 years old	0.268	0.029	-1.434*	0.136	0.155	0.099
At 11 years old	0.264	0.033	-0.267	0.055	0.003	0.071
At 14 years old	-0.179	0.031	0.716	0.125	-0.025	0.072
MEP						
At prenatal	-0.759	0.054	-0.267	0.053	0.439	0.121
At 2 years old	0.496	0.196	0.235	0.083	0.136	0.208
At 5 years old	-0.733	0.058	0.167	0.062	0.180	0.146
At 8 years old	0.880	0.074	-1.342*	0.138	0.236	0.114
At 11 years old	0.111	0.027	0.496	0.081	-0.152	0.098
At 14 years old	-0.193	0.032	-0.211	0.061	-0.075	0.082

*p < 0.05, **p < 0.01.

In the multivariable linear model, the covariates included maternal age at delivery, maternal education, paternal education, and family income.

The bold values indicate significant results (p<0.05).

Pearson correlations were estimated between log-transformed blood hormones and log-transformed urinary phthalate metabolites at 14 years of age (**Supplementary Table S3**, **Supplementary Figure S1-A–D**), 11 years of age (**Supplementary Table S4**), 8 years of age (**Supplementary Table S5**), 5 years of age (**Supplementary Table S6**), 2 years of age (**Supplementary Table S7**), and prenatally (**Supplementary Table S8**). In boys, urinary phthalate metabolite levels at 14 years of age were positively correlated with LH at 14 years of age, and urinary phthalate metabolite levels at 11 years of age were negatively correlated with TT levels at 14 years of age. In girls, urinary phthalate metabolite levels at 11 years of age were negatively correlated with TT levels at 14 years of age, and urinary phthalate metabolite levels at 8 years of age were positively correlated with FSH and LH at 14 years of age.

## 4 Discussion

In the present follow-up study, we examined the association between the pubertal and reproductive development of 14-year-old male and female children and prenatal and environmental exposure to phthalates. Some urinary phthalate metabolite levels correlated with developmental stages in all children. Regarding the development of reproductive organs, we observed evidence of an association between phthalate exposure and reduced utero size.

According to the survey data of the Ministry of Health and Welfare between 2017 and 2020, 14-year-old boys in Taiwan had an average height of 167.7 cm, weight of 62.8 kg, and BMI of 22.2 kg/m^2^, while girls had an average height of 159.6 cm, weight of 53 kg, and BMI of 20.7 kg/m^2^ (33). Our study found that the average height of boys and girls were within the normal growth curve; however, girls who exhibited adult characteristics showed a tendency towards being overweight. During puberty, the levels of GH and IGF-1 increase significantly, leading to a rapid increase in bone and muscle growth (34–36). This growth spurt is further amplified by the increased production of sex steroids, particularly estradiol in females and testosterone in males (34–36). In girls undergoing normal puberty, it is common to observe a physiological increase in body mass index (BMI) and subcutaneous adiposity (37). This phenomenon is likely attributable to the hormonal changes that occur during puberty, including increases in estrogen and other hormones that promote the accumulation of adipose tissue (36–38). In addition, physical activity and participation in sports during adolescence tend to decline, especially among teenage girls (38). This finding does not provide sufficient evidence to infer a causal relationship between height, obesity, and puberty development.

Early puberty can be categorized based on the degree of early onset, which includes premature thelarche (early breast development), premature adrenarche (early pubic hair development), and true precocious puberty (36). Current research indicates that phthalates are associated differently with breast and pubic hair development (36, 39–43). The evidence indicated that MEP is related to early onset of breast development (12, 44), while DEHP and its metabolites may be related to delayed pubic hair development (39–41). Our study results revealed that the MEHP and MEHHP levels of 11-year-old girls were negatively correlated with the Tanner stage at 14 years of age, and the MEP level at 2 years old was associated with a higher Tanner stage at 14 years of age. However, these results cannot establish causality for pubertal delay. The impact of phthalate exposure on pubertal development in girls may have both positive and negative effects.

Animal experiments have demonstrated that exposure to phthalate metabolites before and after birth can lead to decreased TT production, alterations in male reproductive development and function, and delayed puberty (40, 42, 43). However, the available epidemiological data are limited and inconsistent (41, 45–48). A prospective cohort study conducted in Russia has revealed that significantly higher prepubertal urinary concentrations of phthalate metabolites were associated with later pubarche. Specifically, higher concentrations of MiBP, MBzP, and ΣDiNP were associated with a delay in gonadarche (49). In our study, some boys with Tanner stage < 4 exhibited a negative correlation with MBzP exposure at 11 years old, and no correlation was noted between the testicular volume and phthalates. However, at 11 years old, exposure to DEHP, MEHP, MEHHP, and MBzP was negatively associated with testosterone at 14 years of age (**Supplementary Table S3**), similar to the results of Burns et al. (49). Based on these results, in the future, we will consider exploring the association between phthalate exposure during hormone-sensitive pre-and peri-pubertal stages and pubertal development timing among males.

For boys, maternal phthalate exposure probably has adverse effects on the male fetus, with an increased risk of urogenital developmental abnormalities (11). In an early study, marmoset monkeys were treated daily with 0, 100, 500, or 2500 mg/kg DEHP by oral gavage for 65 weeks from weaning (3 months of age) to sexual maturity (18 months) (50). Even in monkeys treated with 2500 mg/kg DEHP throughout the preadolescent period, no histological changes were noted in the testes (50). However, recent animal and human studies have indicated that phthalate exposure results in adverse seminiferous cord effects (51–54), including changes in seminiferous cord development, Sertoli cell function, germ cell death, and multinucleation of germ cells (51, 52).

A meta-analysis concluded that a positive association exists between phthalate exposure and pubertal timing (12). A Swedish cohort study reported that young men (aged 17–20 years) with the highest prenatal levels of the diisononyl phthalate (DiNP) metabolite had a lower testicular volume, and that DEHP was significantly associated with a lower semen volume (54). A cross-sectional Danish study did not find any association between phthalate exposure, testicular growth, and measurable testosterone levels (48). A Spanish study surveyed the association between phthalates and pubertal development in children aged 7–10 years, and the results revealed that child obesity modifies the association of prenatal phthalate exposure with puberty (21).

Some phthalates can disrupt estrogen signaling, increasing estrogen levels, and possibly causing uterine disorders (55, 56). Our previous study detected a significant association between reduced uterine size and increased exposure to MEHP and total DEHP among 11-year-old girls (8); the results also revealed that the uterine size at 14 years of age was negatively related to MEHP, MnBP, MMP, and MBP levels at 8 years and the MBzP level at 14 years of age. For female marmoset monkeys, increased ovarian and uterine weights and elevated blood estradiol levels were observed in higher dosage groups (500 and 2500 mg/kg) (50). However, these increased uterine weights were associated with a large corpus luteum, a common finding in older female marmosets (50). The uterus is a crucial target organ in mammalian development in terms of sexual maturation, and uterine cells respond to estrogen and progesterone for the regulation of endometrial cell proliferation and differentiation (15). Exposure to DEHP can alter the number of endometrial glands and disrupt their structure in rats and mice (17, 18, 56).

Our data also indicated a positive or negative correlation between the hormonal levels of 14-year-old girls and exposure to some phthalates at specific time points (**Supplementary Tables S3–S8**). Furthermore, an association was noted between phthalate exposure at the age of 14 years and hormonal performance in girls. The association between sex hormone levels at 14 years of age and phthalate exposure was the most obvious at the ages of 8 and 11 years, respectively. Another critical window is puberty because the targets of endocrine glands usually show a higher sensitivity to hormones during a specific developmental stage (26, 57). Future studies must investigate the correlation between sex hormones and the uterus. Phthalates are typically stored in adipose tissues. The adipose tissue in girls tends to increase and be redistributed during puberty (22, 26). During this period, girls have a higher likelihood of phthalate accumulation in the adipose tissue; however, this relationship requires further research.

There are limitations to this study. First, the approximately 80% sample size loss to follow-up in this birth cohort study may have caused bias in the exposure–outcome association estimates. A temporal relationship or causality could not be inferred between phthalate exposure and the children’s reproductive developmental indices. Furthermore, EDCs widely exist in human lives; this study lacks an analysis of the effects of co-exposure to EDCs. In the future, animal models or human epidemiological studies with careful exposure assessment will be necessary to determine the causal relationship between individual phthalate exposure and its effects on development. These studies should include adequate sample sizes, standardized exposure protocols, and longitudinal assessments to capture the potential cumulative effects of phthalate exposure over time.

In conclusion, exposure to phthalates at certain time points may be associated with reproductive organ development in girls. Determining whether exposure to phthalates affects later reproductive function requires further investigation. Although we observed that exposure to phthalates at a specific time might be associated with subsequent sexual organ or hormonal effects, this assumption arose from an epidemiological study design. These hypotheses should be proven by randomized studies employing relevant animal models or using a more rigorous epidemiology study design.

## Data availability statement

The original contributions presented in the study are included in the article/**Supplementary Material**. Further inquiries can be directed to the corresponding authors.

## Ethics statement

This study was approved by the Ethics Review Committee of the National Health Research Institutes in Taiwan, and informed written consent was obtained before the commencement of the study from each of the pregnant participants, the guardian, and the children ≥ 6 years of age using suitable language for children’s understanding. Written informed consent to participate in this study was provided by the participants’ legal guardian/next of kin.

## Author contributions

Conceptualization: P-HS. Formal analysis: J-YH. Funding acquisition: None. Methodology: P-HS, S-LW. Writing–original draft: H-PC. Writing–review and editing: P-HS and H-PC. All authors contributed to the article and approved the submitted version.

## References

[1] Dutta S Haggerty DK Rappolee DA Ruden DM. Phthalate exposure and long-term epigenomic consequences: a review. Front Genet (2020) 11:405. doi: 10.3389/fgene.2020.00405 32435260 PMC7218126

[2] Koch HM Lorber M Christensen KL Palmke C Koslitz S Bruning T. Identifying sources of phthalate exposure with human biomonitoring: results of a 48h fasting study with urine collection and personal activity patterns. Int Int J Hyg Environ Health (2013) 216:672–81. doi: 10.1016/j.ijheh.2012.12.002 23333758

[3] Hsieh CJ Chang YH Hu A Chen ML Sun CW Situmorang RF. Personal care products use and phthalate exposure levels among pregnant women. Sci Total Environ (2019) 648:135–43. doi: 10.1016/j.scitotenv.2018.08.149 30114584

[4] Gutierrez-Garcia AK Flores-Kelly JM Ortiz-Rodriguez T Kalixto-Sanchez MA De Leon-Rodriguez A. Phthalates affect the *in vitro* expansion of human hematopoietic stem cell. Cytotechnology (2019) 71:553–61. doi: 10.1007/s10616-019-00300-x PMC6465380 30715687

[5] Wittassek M Angerer J. Phthalates: metabolism and exposure. Int J Androl (2008) 31:131–8. doi: 10.1111/j.1365-2605.2007.00837.x 18070048

[6] Hart R Doherty DA Frederiksen H Keelan JA Hickey M Sloboda D. The influence of antenatal exposure to phthalates on subsequent female reproductive development in adolescence: a pilot study. Reproduction (2014) 147:379–90. doi: 10.1530/REP-13-0331 24025997

[7] Su PH Chen JY Lin CY Chen HY Liao PC Ying TH. Sex steroid hormone levels and reproductive development of eight-year-old children following *in utero* and environmental exposure to phthalates. PloS One (2014) 9:e102788. doi: 10.1371/journal.pone.0102788 25207995 PMC4160173

[8] Su PH Chang CK Lin CY Chen HY Liao PC Hsiung CA. Prenatal exposure to phthalate ester and pubertal development in a birth cohort in central Taiwan: a 12-year follow-up study. Environ Res (2015) 136:324–30. doi: 10.1016/j.envres.2014.10.026 25460653

[9] Xie C Zhao Y Gao L Chen J Cai D Zhang Y. Elevated phthalates' exposure in children with constitutional delay of growth and puberty. Mol Cell Endocrinol (2015) 407:67–73. doi: 10.1016/j.mce.2015.03.006 25770461

[10] Binder AM Corvalan C Calafat AM Ye X Mericq V Pereira A. Childhood and adolescent phenol and phthalate exposure and the age of menarche in latina girls. Environ Health (2018) 17:32. doi: 10.1186/s12940-018-0376-z 29615064 PMC5883544

[11] Pallotti F Pelloni M Gianfrilli D Lenzi A Lombardo F Paoli D. Mechanisms of testicular disruption from exposure to bisphenol a and phtalates. J Clin Med (2020) 9:471–89. doi: 10.3390/jcm9020471 PMC7074154 32046352

[12] Golestanzadeh M Riahi R Kelishadi R. Association of phthalate exposure with precocious and delayed pubertal timing in girls and boys: a systematic review and meta-analysis. Environ Sci Process Impacts (2020) 22:873–94. doi: 10.1039/c9em00512a 32091510

[13] Wang Y Qian H. Phthalates and their impacts on human health. Healthcare (Basel) (2021) 9:603–12. doi: 10.3390/healthcare9050603 PMC8157593 34069956

[14] Eales J Bethel A Galloway T Hopkinson P Morrissey K Short RE. Human health impacts of exposure to phthalate plasticizers: an overview of reviews. Environ Int (2022) 158:106903. doi: 10.1016/j.envint.2021 34601394

[15] Basso CG de Araujo-Ramos AT Martino-Andrade AJ. Exposure to phthalates and female reproductive health: a literature review. Reprod Toxicol (2022) 109:61–79. doi: 10.1016/j.reprotox.2022.02.006 35248714

[16] Tassinari R Tait S Busani L Martinelli A Narciso L Valeri M. Metabolic, reproductive and thyroid effects of bis (2-ethylhexyl) phthalate (DEHP) orally administered to male and female juvenile rats at dose levels derived from children biomonitoring study. Toxicology (2021) 449:152653. doi: 10.1016/j.tox.2020.152653 33309551

[17] Somasundaram DB Manokaran K Selvanesan BC Bhaskaran RS. Impact of di-(2-ethylhexyl) phthalate on the uterus of adult wistar rats. Hum Exp Toxicol (2017) 36:565–72. doi: 10.1177/0960327116657601 27473018

[18] Shanmugam DAS Dhatchanamurthy S Leela KA Bhaskaran RS. Maternal exposure to di (2-ethylhexyl) phthalate (DEHP) causes multigenerational adverse effects on the uterus of F1 and F2 offspring rats. Reprod Toxicol (2023) 115:17–28. doi: 10.1016/j.reprotox.2022.11.006 36435455

[19] Sloboda DM Hickey M Hart R. Reproduction in females: the role of the early life environment. Hum Reprod Update (2011) 17:210–27. doi: 10.1093/humupd/dmq048 20961922

[20] Watkins DJ Sanchez BN Tellez-Rojo MM Lee JM Mercado-Garcia A Blank-Goldenberg C. Phthalate and bisphenol a exposure during *in utero* windows of susceptibility in relation to reproductive hormones and pubertal development in girls. Environ Res (2017) 159:143–51. doi: 10.1016/j.envres.2017.07.051 PMC5623649 28800472

[21] Freire C Castiello F Lopez-Espinosa MJ Beneito A Lertxundi A Jimeno-Romero A. Association of prenatal phthalate exposure with pubertal development in Spanish boys and girls. Environ Res (2022) 213:113606. doi: 10.1016/j.envres.2022 35716812

[22] Mínguez-Alarcón L Burns J Williams PL Korrick SA Lee MM Bather JR. Urinary phthalate metabolite concentrations during four windows spanning puberty (prepuberty through sexual maturity) and association with semen quality among young Russian men. Int J Hyg Environ Health (2022) 243:113977. doi: 10.1016/j.ijheh.2022.113977 35533529 PMC9662187

[23] Nidens N Krönke A Jurkutat A Schlingmann M Poulain T Nüchter M. Associations of prenatal exposure to phthalates and one phthalate substitute with anthropometric measures in early life: results from the German LIFE child cohort study. Best Pract Res Clin Endocrinol Metab (2021) 35:101532. doi: 10.1016/j.beem.2021.101532 34238682

[24] Wen HJ Sie L Su PH Chuang CJ Chen HY Sun CW. Prenatal and childhood exposure to phthalate diesters and sex steroid hormones in 2-, 5-, 8-, and 11-year-old children: a pilot study of the Taiwan maternal and infant cohort study. J Epidemiol (2017) 27:516–23. doi: 10.1016/j.je.2016.10.009 PMC5608604 28576446

[25] Hu J Du G Zhang W Huang H Chen D Wu D. Short-term neonatal/prepubertal exposure of dibutyl phthalate (DBP) advanced pubertal timing and affected hypothalamic kisspeptin/GPR54 expression differently in female rats. Toxicology (2013) 314:65–75. doi: 10.1016/j.tox.2013.09.007 24056307

[26] Abreu AP Kaiser UB. Pubertal development and regulation. Lancet Diabetes Endocrinol (2016) 4:254–64. doi: 10.1016/S2213-8587(15)00418-0 PMC5192018 26852256

[27] Mancini A Magnotto JC Abreu AP. Genetics of pubertal timing. Best Pract Res Clin Endocrinol Metab (2022) 36:101618. doi: 10.1016/j.beem.2022.101618 35183440 PMC8990382

[28] Hertzman C Wiens M. Child development and long-term outcomes: a population health perspective and summary of successful interventions. Soc Sci Med (1996) 43:1083–95. doi: 10.1016/0277-9536(96)00028-7 8890409

[29] Greulich WW Pyle SI. Radiographic atlas of skeletal development of the hand and wrist. 2nd ed. Stanford: Stanford University (1959).

[30] Prader A. Testicular size: assessment and clinical importance. Triangle (1966) 7:240–3. 5920758

[31] Badouraki M Christoforidis A Economou I Dimitriadis AS Katzos G. Evaluation of pelvic ultrasonography in the diagnosis and differentiation of various forms of sexual precocity in girls. Ultrasound Obstet Gynecol (2008) 32:819–27. doi: 10.1002/uog.6148 18951545

[32] Tanner JM. Growth at adolescence. 2nd ed. Oxford: Blackwell (1962).

[33] Department of Statistics, Ministry of Health and Welfare Health and Gender Statistics. Indicators - height, weight, body mass index (2023). Available at: https://dep.mohw.gov.tw/DOS/cp-5339-59467-113.html (Accessed March 29, 2023).

[34] Mauras N. Growth hormone and sex steroids. interactions in puberty. Endocrinol Metab Clin North Am (2001) 30:529–44. doi: 10.1016/s0889-8529(05)70200-0 11571929

[35] Marcovecchio ML Chiarelli F. Obesity and growth during childhood and puberty. World Rev Nutr Diet (2013) 106:135–41. doi: 10.1159/000342545 23428692

[36] Shalitin S Gat-Yablonski G. Associations of obesity with linear growth and puberty. Horm Res Paediatr (2022) 95:120–36. doi: 10.1159/000516171 34130293

[37] Solorzano CMB McCartney CR. Obesity and the pubertal transition in girls and boys. Reproduction (2010) 140:399–410. doi: 10.1530/REP-10-0119 20802107 PMC2931339

[38] Alberga AS Sigal RJ Goldfield G Prud'Homme D Kenny GP. Overweight and obese teenagers: why is adolescence a critical period? Pediatr Obes (2012) 7:261–73. doi: 10.1111/j.2047-6310.2011.00046.x 22461384

[39] Wolff MS Teitelbaum SL McGovern K Windham GC Pinney SM Galvez M. Phthalate exposure and pubertal development in a longitudinal study of US girls. Hum Reprod (2014) 29:1558–66. doi: 10.1093/humrep/deu081 PMC4059333 24781428

[40] Frederiksen H Sørensen K Mouritsen A Aksglaede L Hagen CP Petersen JH. High urinary phthalate concentration associated with delayed pubarche in girls. Int J Androl (2012) 35:216–26. doi: 10.1111/j.1365-2605.2012.01260.x 22428786

[41] Shi H Cao Y Shen Q Zhao Y Zhang Z Zhang Y. Association between urinary phthalates and pubertal timing in Chinese adolescents. J Epidemiol (2015) 25:574–82. doi: 10.2188/jea.JE20140205 PMC4549609 26212725

[42] Bao AM Man XM Guo XJ Dong HB Wang FQ Sun H. Effects of din-butyl phthalate on male rat reproduction following pubertal exposure. Asian J Androl (2011) 13:702–9. doi: 10.1038/aja.2011.76 PMC3739579 21841806

[43] Foster PM Mylchreest E Gaido KW Sar M. Effects of phthalate esters on the developing reproductive tract of male rats. Hum Reprod Update (2001) 7:231–5. doi: 10.1093/humupd/7.3.231 11392369

[44] Rivera-Núñez Z Kinkade CW Zhang Y Rockson A Bandera EV Llanos AA. Phenols, parabens, phthalates and puberty: a systematic review of synthetic chemicals commonly found in personal care products and girls’ pubertal development. Curr Environ Health Rep (2022) 9:517–34. doi: 10.1007/s40572-022-00366-4 PMC9742306 35867279

[45] Moore RW Rudy TA Lin TM Ko K Peterson RE. Abnormalities of sexual development in male rats with *in utero* and lactational exposure to the antiandrogenic plasticizer di (2-ethylhexyl) phthalate. Environ Health Perspect (2001) 109:229–37. doi: 10.1289/ehp.01109229 PMC1240240 11333183

[46] Zhang Y Cao Y Shi H Jiang X Zhao Y Fang X. Could exposure to phthalates speed up or delay pubertal onset and development? a 1.5-year follow-up of a school-based population. Environ Int (2015) 83:41–9. doi: 10.1016/j.envint.2015.06.005 26073845

[47] Ferguson KK Peterson KE Lee JM Merc ado-Garcia A Blank-Goldenberg C Tellez-Rojo MM. Prenatal and peripubertal phthalates and bisphenol a in relation to sex hormones and puberty in boys. Reprod Toxicol (2014) 47:70–6. doi: 10.1016/j.reprotox.2014.06.002 PMC4117729 24945889

[48] Mieritz MG Frederiksen H Sorensen K Aksglaede L Mouritsen A Hagen CP. Urinary phthalate excretion in 555 healthy Danish boys with and without pubertal gynaecomastia. Int J Androl (2012) 35:227–35. doi: 10.1111/j.1365-2605.2012.01279.x 22612475

[49] Burns JS Sergeyev O Lee MM Williams PL Mínguez-Alarcón L Plaku-Alakbarova B. Associations of prepubertal urinary phthalate metabolite concentrations with pubertal onset among a longitudinal cohort of boys. Environ Res (2022) 212:113218. doi: 10.1016/j.envres.2022.113218 35390299 PMC9310051

[50] Tomonari Y Kurata Y David RM Gans G Kawasuso T Katoh M. Effect of di (2-ethylhexyl) phthalate (DEHP) on genital organs from juvenile common marmosets: i. morphological and biochemical investigation in 65-week toxicity study. J Toxicol Environ Health A (2006) 69:1651–72. doi: 10.1080/15287390600630054 16854791

[51] Spade DJ Dere E Hall SJ Schorl C Freiman RN Boekelheide K. All-trans retinoic acid disrupts development in ex vivo cultured fetal rat testes. I: altered seminiferous cord maturation and testicular cell fate. Toxicol Sci (2019) 167:546–58. doi: 10.1093/toxsci/kfy260 PMC6358251 30329139

[52] Li H Spade DJ. Reproductive tocicology: environmental exposures, fetal testis development and function: phthalates and beyond. Reproduction (2021) 162:F147–67. doi: 10.1530/REP-20-0592 PMC8497445 34314370

[53] Swan SH Main KM Liu F Stewart SL Kruse RL Calafat AM. Decrease in anogenital distance among male infants with prenatal phthalate exposure. Environ Health Perspect (2005) 113:1056–61. doi: 10.1289/ehp.8100 PMC1280349 16079079

[54] Axelsson J Rylander L Rignell-Hydbom A Jönsson BA Lindh CH Giwercman A. Phthalate exposure and reproductive parameters in young men from the general Swedish population. Environ Int (2015) 85:54–60. doi: 10.1016/j.envint.2015.07.005 26318515

[55] Cavanagh JAE Trought K Mitchell C Northcott G Tremblay LA. Assessment of endocrine disruption and oxidative potential of bisphenol-a, triclosan, nonylphenol, diethylhexyl phthalate, galaxolide, and carbamazepine, common contaminants of municipal biosolids. Toxicol In Vitro (2018) 48:342–9. doi: 10.1016/j.tiv.2018.02.003 29427707

[56] Richardson KA Hannon PR Johnson-Walker YJ Myint MS Flaws JA Nowak RA. Di (2-ethylhexyl) phthalate (DEHP) alters proliferation and uterine gland numbers in the uteri of adult exposed mice. Reprod Toxicol (2018) 77:70–9. doi: 10.1016/j.reprotox.2018.01.006 29458081

[57] Gore AC Chappell VA Fenton SE Flaws JA Nadal A Prins GS. EDC-2: the endocrine society's second scientific statement on endocrine-disrupting chemicals. Endocr Rev (2015) 36:E1–E150. doi: 10.1210/er.2015-1010 26544531 PMC4702494

