# Supplementary material for: Phthalates exposure and pubertal development in a 15-year follow-up birth cohort study in Taiwan
Source: Front Endocrinol (Lausanne). 2023 May 23;14:1065918. doi: 10.3389/fendo.2023.1065918 (PMC10242106; doi:10.3389/fendo.2023.1065918)
Supplement: Supplementary file 1 [file DataSheet_1.docx]

Supplementary Table S1 Baseline (2000–2001) characteristics of mothers stratified by completion status during follow-up (N = 220)

| Variable | Follow-up still age 14 (n=90) | Lost to follow up  (n=130) | p value |
| --- | --- | --- | --- |
| Maternal age at delivery | 30.33±3.66 | 28.40±4.16 | 0.0005 |
| Maternal education |  |  | 0.1675 |
| <12 years | 72 (80.0%) | 113 (86.9%) |  |
| ≧12 years | 18 (20.0%) | 17 (13.1%) |  |
| Parternal education |  | Missing=1 | 0.6308 |
| <12 years | 68 (75.6%) | 101 (77.7%) |  |
| ≧12 years | 22 (24.4%) | 28 (21.5%) |  |
| Family income per year | Missing=1 | Missing=1 | 0.0823 |
| <600,000 NTDs | 30 (33.3%) | 63 (48.5%) |  |
| ≧600,000 NTDs | 59 (65.6%) | 66 (50.8%) |  |
| Smoking during regency |  |  | 0.2715 |
| No | 90 (100.0%) | 127 (97.7%) |  |
| Yes | 0 (0.0%) | 3 (2.3%) |  |
| Maternal urinary phthalate metabolite (μg/g creatinine) | Geometric mean  (95% CI) | Geometric mean  (95% CI) |  |
| MEHP | 18.56 (15.21-22.64) | 17.98 (15.27-21.18) | 0.8072 |
| MEHHP | 7.09 (4.69-10.72) | 8.07 (5.83-11.16) | 0.6234 |
| MEOHP | 13.95 (10.31-18.88) | 11.03 (8.03-15.16) | 0.2908 |
| ΣDEHP | 51.44 (41.64-63.53) | 55.16 (46.45-65.50) | 0.6097 |
| MBP | 70.82 (58.10-86.33) | 73.27 (62.02-86.56) | 0.7955 |
| MBzP | 14.69 (12.22-17.66) | 16.77 (14.60-19.28) | 0.2471 |
| MMP | 51.22 (40.61-64.59) | 53.62 (44.74-64.25) | 0.7552 |
| MEP | 61.36 (50.86-74.03) | 68.92 (58.87-80.70) | 0.3492 |

Values are presented as mean±standard deviation, n(%), and 95% Confidence Interval(C.I.)

Supplementary Table S2 the Pearson correlation between puberty development at 14 years old and log-transformed urinary phthalate metabolite at prenatal, 2, 5, 8, 11, and 14 years old.

|  | Puberty development **at 14 years old** | | |
| --- | --- | --- | --- |
| Log-transformed value of urinary phthalate metabolite | Boys: Testis volume (ml) | Girls: Uterus volume (cm3) | Girls: Ovary volume (cm3) |
| MEHP |  |  |  |
| At prenatal | 0.025 | -0.250 | -0.001 |
| At 2 years old | -0.219 | -0.200 | -0.099 |
| At 5 years old | 0.130 | 0.093 | 0.154 |
| At 8 years old | -0.045 | **-0.314*** | 0.108 |
| At 11 years old | -0.085 | 0.055 | 0.047 |
| At 14 years old | -0.145 | -0.173 | 0.041 |
| MEHHP |  |  |  |
| At prenatal | 0.152 | -0.067 | -0.019 |
| At 2 years old | -0.116 | -0.297 | 0.018 |
| At 5 years old | 0.058 | -0.096 | 0.116 |
| At 8 years old | 0.108 | -0.145 | 0.217 |
| At 11 years old | -0.117 | -0.006 | 0.150 |
| At 14 years old | -0.242 | -0.116 | 0.142 |
| MEOHP |  |  |  |
| At prenatal | 0.170 | -0.078 | -0.192 |
| At 2 years old | -0.145 | **-0.367*** | -0.131 |
| At 5 years old | 0.044 | -0.045 | 0.168 |
| At 8 years old | 0.069 | -0.173 | 0.174 |
| At 11 years old | -0.084 | 0.027 | -0.039 |
| At 14 years old | -0.296 | -0.187 | 0.015 |
| ΣDEHP |  |  |  |
| At prenatal | 0.071 | -0.214 | -0.072 |
| At 2 years old | -0.158 | -0.324 | -0.045 |
| At 5 years old | 0.092 | -0.052 | 0.133 |
| At 8 years old | 0.060 | -0.218 | 0.144 |
| At 11 years old | -0.120 | 0.023 | 0.101 |
| At 14 years old | -0.249 | -0.152 | 0.129 |
| MnBP |  |  |  |
| At prenatal | 0.149 | **-0.305*** | 0.032 |
| At 2 years old | 0.012 | -0.088 | -0.061 |
| At 5 years old | -0.184 | 0.083 | -0.095 |
| At 8 years old | 0.079 | **-0.353*** | 0.002 |
| At 11 years old | 0.269 | 0.129 | -0.010 |
| At 14 years old | -0.067 | 0.158 | -0.087 |
| MBzP |  |  |  |
| At prenatal | -0.149 | -0.014 | 0.089 |
| At 2 years old | 0.058 | 0.083 | -0.039 |
| At 5 years old | 0.242 | 0.288 | 0.098 |
| At 8 years old | 0.107 | -0.153 | 0.081 |
| At 11 years old | -0.063 | -0.070 | 0.076 |
| At 14 years old | -0.230 | **-0.347*** | -0.028 |
| MMP |  |  |  |
| At prenatal | -0.154 | **-0.464**** | 0.037 |
| At 2 years old | 0.144 | 0.063 | -0.094 |
| At 5 years old | 0.073 | -0.052 | 0.065 |
| At 8 years old | 0.117 | **-0.277*** | 0.088 |
| At 11 years old | 0.082 | -0.056 | 0.067 |
| At 14 years old | -0.131 | 0.188 | 0.039 |
| MEP |  |  |  |
| At prenatal | -0.131 | -0.074 | 0.259 |
| At 2 years old | 0.243 | 0.030 | 0.027 |
| At 5 years old | -0.225 | 0.065 | 0.101 |
| At 8 years old | 0.252 | -0.259 | 0.154 |
| At 11 years old | 0.055 | 0.147 | -0.135 |
| At 14 years old | -0.085 | -0.061 | -0.147 |

* p<0.05, ** p<0.01

Supplementary Table S3. The Pearson correlation between log-transformed blood hormone and log-transformed urinary phthalate metabolite at 14 years old.

|  | Log-transformed value of blood hormone **at 14 years old** | | | | | |
| --- | --- | --- | --- | --- | --- | --- |
| Log-transformed value of urinary phthalate metabolite **at 14 years old** | Estradiol | Testosterone | Progesterone | FSH | LH | SHBG |
| In boys |  |  |  |  |  |  |
| MEHP | 0.108 | -0.030 | 0.245 | 0.049 | **0.464**** | 0.122 |
| MEHHP | 0.136 | -0.125 | 0.128 | 0.163 | 0.291 | -0.062 |
| MEOHP | 0.032 | -0.108 | 0.077 | 0.104 | 0.277 | 0.084 |
| DEHP | 0.101 | -0.103 | 0.143 | 0.138 | **0.329*** | 0.026 |
| MnBP | 0.050 | 0.077 | 0.127 | 0.037 | 0.237 | -0.025 |
| MBzP | -0.159 | -0.045 | -0.079 | 0.086 | 0.124 | 0.258 |
| MMP | -0.062 | -0.050 | 0.222 | -0.010 | 0.050 | -0.244 |
| MEP | -0.042 | -0.059 | 0.133 | 0.135 | **0.415**** | 0.014 |
| In girls |  |  |  |  |  |  |
| MEHP | -0.016 | 0.162 | -0.107 | 0.263 | -0.111 | 0.139 |
| MEHHP | 0.018 | -0.035 | **-0.310*** | 0.246 | -0.016 | 0.020 |
| MEOHP | -0.018 | 0.027 | -0.160 | 0.197 | -0.067 | 0.152 |
| DEHP | 0.014 | 0.016 | -0.265 | 0.282* | -0.045 | 0.096 |
| MnBP | 0.119 | -0.125 | -0.239 | 0.263 | 0.170 | 0.211 |
| MBzP | 0.008 | 0.126 | 0.056 | 0.186 | -0.036 | -0.029 |
| MMP | -0.126 | **-0.292*** | -0.260 | 0.110 | 0.047 | -0.100 |
| MEP | -0.201 | -0.068 | -0.047 | -0.028 | -0.153 | 0.035 |

* p<0.05, ** p<0.01, LH, Luteinizing Hormone; FSH, Follicle-stimulating hormone; SHBG, Sex Hormone-Binding Globulin

Supplementary Table S4. The Pearson correlation between log-transformed blood hormone at 14 years old and log-transformed urinary phthalate metabolite at 11 years old.

|  | Log-transformed value of blood hormone **at 14 years old** | | | | | |
| --- | --- | --- | --- | --- | --- | --- |
| Log-transformed value of urinary phthalate metabolite **at 11 years old** | Estradiol | Testosterone | Progesterone | FSH | LH | SHBG |
| In boys |  |  |  |  |  |  |
| MEHP | -0.074 | **-0.335*** | 0.263 | -0.089 | 0.301 | -0.165 |
| MEHHP | -0.131 | **-0.414**** | 0.115 | -0.124 | 0.053 | -0.170 |
| MEOHP | -0.032 | -0.265 | 0.259 | -0.104 | 0.188 | -0.284 |
| DEHP | -0.115 | **-0.419**** | 0.168 | -0.108 | 0.141 | -0.217 |
| MnBP | 0.240 | -0.098 | 0.229 | -0.299 | -0.036 | -0.056 |
| MBzP | -0.128 | **-0.409**** | -0.018 | -0.198 | -0.179 | -0.046 |
| MMP | -0.031 | -0.149 | 0.050 | 0.259 | 0.100 | 0.160 |
| MEP | 0.096 | -0.259 | 0.083 | 0.097 | -0.043 | -0.150 |
| In girls |  |  |  |  |  |  |
| MEHP | -0.201 | -0.182 | -0.094 | 0.217 | -0.017 | 0.107 |
| MEHHP | -0.258 | **-0.397**** | **-0.290*** | **0.282*** | -0.001 | 0.022 |
| MEOHP | -0.257 | **-0.360*** | -0.146 | 0.142 | 0.019 | -0.101 |
| DEHP | -0.260 | **-0.379**** | -0.244 | 0.263 | 0.009 | 0.023 |
| MnBP | 0.121 | -0.046 | -0.056 | -0.037 | 0.044 | -0.097 |
| MBzP | -0.049 | -0.042 | -0.057 | 0.099 | -0.057 | -0.038 |
| MMP | -0.111 | 0.059 | **-0.341*** | 0.219 | 0.117 | -0.049 |
| MEP | 0.150 | 0.059 | 0.159 | 0.005 | 0.245 | -0.045 |

* p<0.05, ** p<0.01, LH, Luteinizing Hormone; FSH, Follicle-stimulating hormone; SHBG, Sex Hormone-Binding Globulin

Supplementary Table S5. The Pearson correlation between log-transformed blood hormone at 14 years old and log-transformed urinary phthalate metabolite at 8 years old.

|  | Log-transformed value of blood hormone **at 14 years old** | | | | | |
| --- | --- | --- | --- | --- | --- | --- |
| Log-transformed value of urinary phthalate metabolite **at 8 years old** | Estradiol | Testosterone | Progesterone | FSH | LH | SHBG |
| In boys |  |  |  |  |  |  |
| MEHP | -0.050 | -0.067 | 0.090 | -0.059 | 0.133 | -0.172 |
| MEHHP | -0.009 | -0.145 | 0.226 | 0.028 | 0.085 | -0.236 |
| MEOHP | -0.078 | -0.113 | 0.198 | 0.017 | 0.105 | -0.197 |
| DEHP | -0.027 | -0.108 | 0.210 | -0.003 | 0.106 | -0.224 |
| MnBP | 0.096 | -0.040 | 0.240 | -0.129 | -0.058 | -0.120 |
| MBzP | 0.029 | -0.006 | 0.180 | -0.018 | -0.174 | -0.311 |
| MMP | 0.173 | -0.125 | -0.149 | -0.272 | -0.196 | -0.060 |
| MEP | 0.283 | -0.028 | 0.028 | -0.055 | -0.253 | -0.147 |
| In girls |  |  |  |  |  |  |
| MEHP | -0.064 | 0.250 | -0.020 | 0.271 | 0.229 | -0.181 |
| MEHHP | -0.031 | 0.226 | 0.015 | **0.306*** | **0.321*** | -0.197 |
| MEOHP | 0.009 | 0.227 | 0.057 | **0.287*** | **0.319*** | -0.202 |
| DEHP | -0.098 | 0.235 | -0.007 | **0.317*** | **0.287*** | -0.178 |
| MnBP | -0.157 | 0.273 | -0.081 | **0.379**** | 0.273 | -0.057 |
| MBzP | 0.036 | 0.175 | 0.051 | 0.134 | 0.161 | -0.070 |
| MMP | -0.119 | 0.297 | -0.092 | 0.281 | 0.234 | -0.030 |
| MEP | 0.122 | **0.365*** | 0.116 | 0.188 | 0.263 | 0.050 |

* p<0.05, ** p<0.01, FSH, Follicle-stimulating hormone; LH, Luteinizing Hormone; SHBG, Sex Hormone-Binding Globulin

Supplementary Table S6. The Pearson correlation between log-transformed blood hormone at 14 years old and log-transformed urinary phthalate metabolite at 5 years old.

|  | Log-transformed value of blood hormone **at 14 years old** | | | | | |
| --- | --- | --- | --- | --- | --- | --- |
| Log-transformed value of urinary phthalate metabolite **at 5 years old** | Estradiol | Testosterone | Progesterone | FSH | LH | SHBG |
| In boys |  |  |  |  |  |  |
| MEHP | -0.258 | -0.175 | -0.096 | -0.111 | 0.154 | -0.091 |
| MEHHP | -0.059 | -0.046 | -0.043 | -0.024 | -0.047 | -0.236 |
| MEOHP | -0.236 | -0.111 | -0.108 | 0.024 | -0.018 | -0.185 |
| DEHP | -0.172 | -0.095 | -0.067 | -0.056 | -0.023 | -0.240 |
| MnBP | 0.083 | 0.211 | -0.192 | -0.053 | 0.171 | 0.145 |
| MBzP | -0.337 | -0.240 | -0.279 | 0.150 | -0.128 | -0.304 |
| MMP | -0.085 | -0.134 | -0.186 | -0.218 | 0.149 | 0.034 |
| MEP | -0.319 | -0.097 | **-0.364*** | 0.075 | -0.004 | 0.032 |
| In girls |  |  |  |  |  |  |
| MEHP | 0.101 | 0.044 | 0.091 | -0.086 | -0.020 | -0.177 |
| MEHHP | 0.074 | 0.205 | 0.279 | -0.077 | 0.123 | -0.373 |
| MEOHP | 0.047 | 0.133 | 0.172 | -0.080 | 0.064 | **-0.346*** |
| DEHP | 0.072 | 0.177 | 0.245 | -0.096 | 0.084 | **-0.346*** |
| MnBP | -0.065 | -0.061 | 0.016 | 0.146 | 0.286 | 0.037 |
| MBzP | 0.113 | -0.001 | 0.243 | **-0.440**** | -0.285 | 0.032 |
| MMP | 0.082 | 0.010 | 0.131 | 0.040 | 0.128 | -0.108 |
| MEP | **0.416*** | **0.358*** | 0.374* | -0.134 | **0.351*** | 0.006 |

* p<0.05, ** p<0.01, FSH, Follicle-stimulating hormone; LH, Luteinizing Hormone; SHBG, Sex Hormone-Binding Globulin

Supplementary Table S7. The Pearson correlation between log-transformed blood hormone at 14 years old and log-transformed urinary phthalate metabolite at 2 years old.

|  | Log-transformed value of blood hormone **at 14 years old** | | | | | |
| --- | --- | --- | --- | --- | --- | --- |
| Log-transformed value of urinary phthalate metabolite **at 2 years old** | Estradiol | Testosterone | Progesterone | FSH | LH | SHBG |
| In boys |  |  |  |  |  |  |
| MEHP | 0.291 | **0.502**** | 0.140 | -0.044 | 0.359 | 0.261 |
| MEHHP | -0.073 | 0.151 | 0.135 | 0.267 | 0.212 | 0.142 |
| MEOHP | -0.148 | 0.014 | 0.277 | 0.230 | 0.178 | 0.107 |
| DEHP | -0.061 | 0.173 | 0.184 | 0.258 | 0.226 | 0.142 |
| MnBP | -0.139 | 0.028 | 0.108 | -0.042 | 0.071 | -0.066 |
| MBzP | 0.081 | -0.043 | 0.085 | -0.045 | -0.167 | -0.129 |
| MMP | 0.059 | 0.097 | -0.075 | 0.066 | -0.357 | -0.115 |
| MEP | -0.020 | -0.085 | -0.177 | -0.217 | -0.311 | 0.257 |
| In girls |  |  |  |  |  |  |
| MEHP | -0.156 | -0.288 | -0.319 | 0.182 | -0.279 | -0.202 |
| MEHHP | 0.103 | 0.005 | -0.066 | 0.058 | 0.027 | -0.225 |
| MEOHP | 0.062 | 0.026 | -0.074 | 0.147 | 0.034 | -0.172 |
| DEHP | 0.057 | -0.027 | -0.115 | 0.110 | 0.004 | -0.229 |
| MnBP | -0.039 | -0.073 | -0.196 | 0.072 | 0.058 | -0.170 |
| MBzP | -0.052 | 0.076 | -0.210 | -0.061 | 0.056 | 0.150 |
| MMP | -0.103 | -0.023 | **-0.398*** | -0.028 | 0.027 | -0.090 |
| MEP | -0.056 | -0.006 | -0.006 | -0.200 | -0.324 | 0.050 |

* p<0.05, ** p<0.01, FSH, Follicle-stimulating hormone; LH, Luteinizing Hormone; SHBG, Sex Hormone-Binding Globulin

Supplementary Table S8. The Pearson correlation between log-transformed blood hormone at 14 years old and log-transformed urinary phthalate metabolite at prenatal years old.

|  | Log-transformed value of blood hormone **at 14 years old** | | | | | |
| --- | --- | --- | --- | --- | --- | --- |
| Log-transformed value of urinary phthalate metabolite **at prenatal** | Estradiol | Testosterone | Progesterone | FSH | LH | SHBG |
| In boys |  |  |  |  |  |  |
| MEHP | -0.199 | -0.030 | -0.243 | -0.051 | -0.252 | 0.027 |
| MEHHP | -0.102 | 0.312 | -0.034 | -0.016 | -0.094 | -0.032 |
| MEOHP | -0.044 | 0.244 | 0.052 | -0.246 | -0.234 | 0.050 |
| DEHP | -0.119 | 0.186 | -0.112 | -0.130 | -0.211 | 0.049 |
| MnBP | -0.016 | -0.130 | -0.091 | **-0.373*** | -0.058 | -0.107 |
| MBzP | -0.078 | 0.067 | -0.155 | -0.036 | -0.042 | 0.015 |
| MMP | -0.052 | -0.114 | -0.147 | -0.227 | 0.048 | -0.060 |
| MEP | -0.172 | -0.190 | -0.183 | 0.039 | -0.158 | -0.126 |
| In girls |  |  |  |  |  |  |
| MEHP | 0.133 | 0.092 | -0.069 | 0.187 | 0.028 | -0.013 |
| MEHHP | 0.064 | -0.083 | 0.108 | -0.164 | -0.162 | -0.170 |
| MEOHP | -0.096 | -0.046 | -0.011 | 0.021 | -0.218 | -0.184 |
| DEHP | 0.031 | -0.028 | -0.048 | 0.072 | -0.163 | -0.087 |
| MnBP | 0.058 | -0.017 | 0.041 | 0.037 | -0.059 | -0.197 |
| MBzP | -0.116 | **-0.307*** | -0.172 | -0.019 | -0.247 | -0.180 |
| MMP | 0.014 | 0.035 | -0.025 | -0.039 | -0.104 | -0.232 |
| MEP | 0.085 | 0.247 | 0.137 | -0.063 | -0.195 | -0.228 |

* p<0.05, ** p<0.01, FSH, Follicle-stimulating hormone; LH, Luteinizing Hormone; SHBG, Sex Hormone-Binding Globulin

**In boys**

Y=ln(Estradiol)


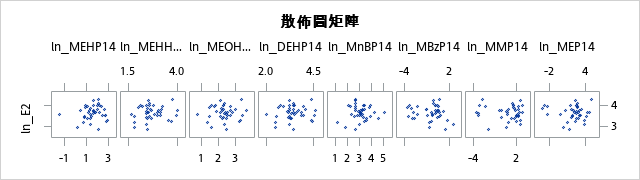


Y=ln(Testosterone)


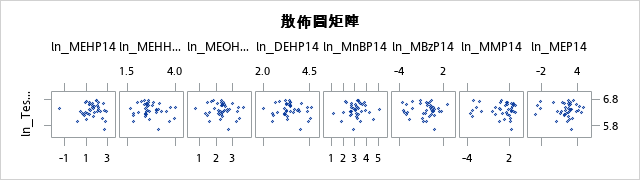


Y=ln(Progesterone)


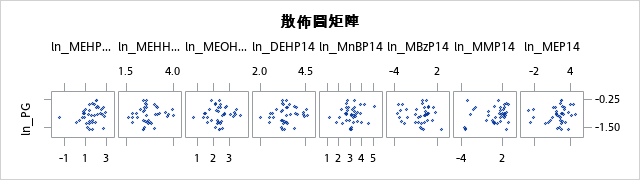


Supplementary Figure S1-(A). The correlation between Log-transformed blood hormone with Log-transformed urinary phthalate metabolite at 14 years old.

**In boys**

Y=ln(FSH)


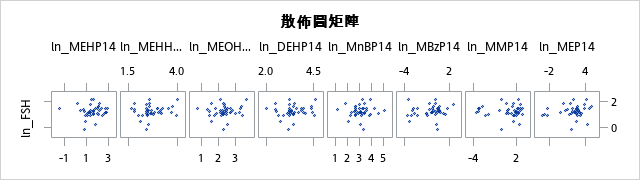


Y=ln(LH)


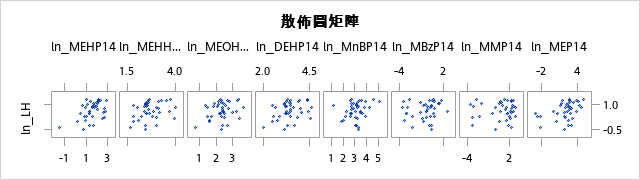


Y=ln(SHBG)


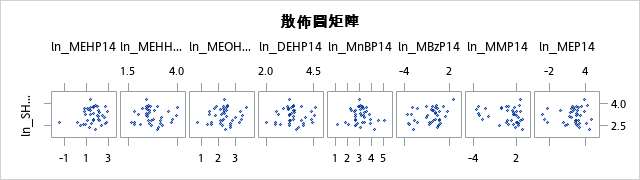


Supplementary Figure S1-(B). The correlation between Log-transformed blood hormone with Log-transformed urinary phthalate metabolite at 14 years old

**In girls**

Y=ln(ESTRADIOL)


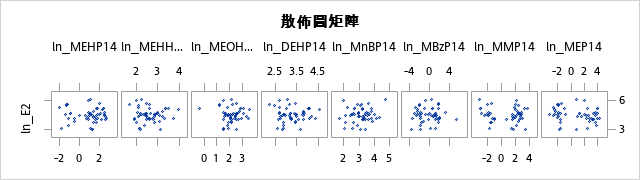


Y=ln(Testosterone)


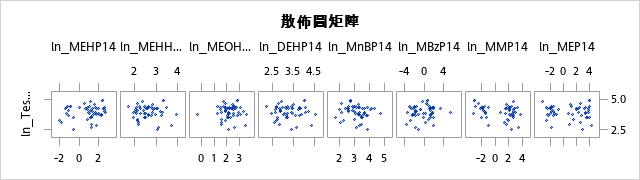


Y=ln(PG)


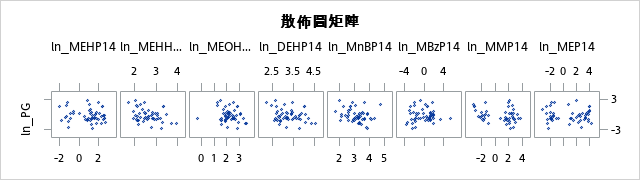


Supplementary Figure S1-(C). The correlation between Log-transformed blood hormone with Log-transformed urinary phthalate metabolite at 14 years old

**In girls**

Y=ln(FSH)


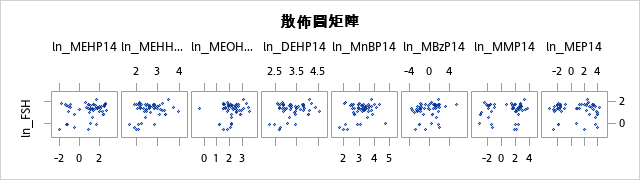


Y=ln(LH)


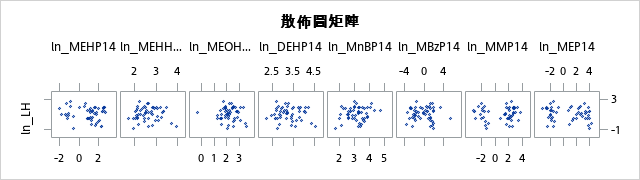


Y=ln(SHBG)


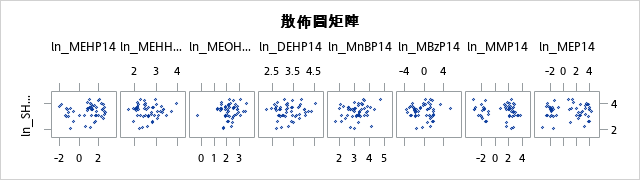


Supplementary Figure S1-(D). The correlation between Log-transformed blood hormone with Log-transformed urinary phthalate metabolite at 14 years old
